# The association between meteorological factors and road traffic injuries: a case analysis from Shantou city, China

**DOI:** 10.1038/srep37300

**Published:** 2016-11-17

**Authors:** Jinghong Gao, Xiaojun Chen, Alistair Woodward, Xiaobo Liu, Haixia Wu, Yaogui Lu, Liping Li, Qiyong Liu

**Affiliations:** 1 State Key Laboratory of Infectious Disease Prevention and Control, Collaborative Innovation Center for Diagnosis and Treatment of Infectious Diseases, National Institute for Communicable Disease Control and Prevention, Chinese Center for Disease Control and Prevention, Beijing, China; 2 Injury Prevention Research Center, Shantou University Medical College, No. 22 Xinling Road, Shantou, Guangdong, 515041, China; 3 First Affiliated Hospital of Shantou University Medical College, Guangdong, China; 4 School of Population Health, University of Auckland, Private Bag 92019, Auckland, New Zealand

## Abstract

Few studies examined the associations of meteorological factors with road traffic injuries (RTIs). The purpose of the present study was to quantify the contributions of meteorological factors to RTI cases treated at a tertiary level hospital in Shantou city, China. A time-series diagram was employed to illustrate the time trends and seasonal variation of RTIs, and correlation analysis and multiple linear regression analysis were conducted to investigate the relationships between meteorological parameters and RTIs. RTIs followed a seasonal pattern as more cases occurred during summer and winter months. RTIs are positively correlated with temperature and sunshine duration, while negatively associated with wind speed. Temperature, sunshine hour and wind speed were included in the final linear model with regression coefficients of 0.65 (t = 2.36, *P* = 0.019), 2.23 (t = 2.72, *P* = 0.007) and −27.66 (t = −5.67, *P* < 0.001), respectively, accounting for 19.93% of the total variation of RTI cases. The findings can help us better understand the associations between meteorological factors and RTIs, and with potential contributions to the development and implementation of regional level evidence-based weather-responsive traffic management system in the future.

Road traffic injuries (RTIs) are traditionally regarded as unforeseen and unexpected events involving motor vehicles, but are now one of the biggest public health threats worldwide^1^,^2^. According to the World Health Organization, 1.24 million people die and an additional 50 million are injured annually as a result of RTIs^3^,^4^. In 2013, road injuries in total led to 1.4 (95% CI: 1.29–1.19) million deaths and 73.25 (95% CI: 66.86–78.67) million disability-adjusted life years (DALYs) around the world^5^,^6^. In 2004, an estimated 85% of RTI-related deaths and 90% of DALYs caused by RTIs occurred in low- and middle-income countries^7^,^8^. Globally, studies suggest that injuries, disabilities and deaths resulted from RTIs are costing 1%, 1.5% and 2% of the gross national product of low-, middle- and high-income countries, respectively (amounting to losses of about 518 billion dollars yearly)^9^,^10^,^11^. If appropriate actions are not taken, RTIs are expected to move from the ninth (in 1998) to be the third leading contributor to annual DALYs lost by the end of 2020, and to be the fifth leading cause of death by the year 2030, bringing about approximately 2.4 million deaths around the world, greater than the numbers attributed to malaria, tuberculosis and HIV/AIDS^1^,^7^,^11^,^12^.

In China, with rapid motorization fueled by social development and economic growth over the last three decades, the number of motor vehicles has increased up to about 264 million—the second largest number of vehicles only after America^13^,^14^, while RTI-related mortality has increased 81% from 1987 to 2006, and the direct economic losses due to RTIs accounted for around 1–3% of China’s annual gross domestic product^8^,^15^,^16^. RTIs have become the first cause of death for people up to the age of 45 years and the leading cause of working-life years lost in China^15^,^17^,^18^. In 2010, the mortality rate of RTIs was 20.5 per 100,000 people in China, which is higher than the figure in developed countries (e.g. 5.2 per 100,000 in Japan, and 4.7 per 100,000 in Germany), and even higher than that in many developing countries (e.g. 17.7 per 100,000 in Indonesia, and 18.9 per 100,000 in India)^13^,^19^. According to the *National statistics yearbook of China*, although RTI-related deaths have decreased slightly after 2004 (Fig. 1), the total burden of losses is still very high (http://www.stats.gov.cn/tjsj/ndsj/). For instance, there were 224,327 injuries and 59,997 deaths caused by 204,196 road traffic accidents in 2012, which resulted in over 7,278 million dollars in direct property losses^20^.

Since the famous “Haddon matrix model” consisted of three sets of interactive factors, namely human, vehicle and environment factors, was introduced by William Haddon in 1968, contributions of human factors, road environment and vehicle design to the occurrence and severity of RTIs have been closely investigated. However, relatively limited research has been performed specifically on the associations between meteorological factors and RTIs, especially in low- and middle-income countries, where RTIs have increased so quickly because of rapid urbanization and motorization^16^,^21^. According to the conceptual framework of the interplay and interaction between RTIs and meteorological, human, vehicle and environment factors (Fig. 2), weather conditions may play a role, both directly and indirectly, in the frequency and severity of RTIs^21^,^22^,^23^. In Calgary and Edmonton, Canada, according to the police reports of traffic accidents during 1979–1983, nearly 90% were attributable to human factors, and “slippery road” due to rainfall, snow or ice was the most frequently cited environmental condition, which was the main contributing factor in 9% of the cases^24^. In 2001, the Federal Highway Administration indicated that weather contributed to over 22% of the total traffic crashes^25^. In Washington, USA, the contribution of weather and related interactions to the likelihood of crash occurrence was about 19%^26^. As a contributing factor, it is estimated that weather conditions can explain 5% of the variation in injury crashes^27^,^28^, approximately 28% of all highway crashes, and 19% of all RTI-related fatalities^29^.

It worth noticing, that the specific figures of the contributions of meteorological factors to RTIs differ greatly from study to study conducted in different countries or regions due to local factors, including climatic environments, socioeconomic status and road user type^26^,^27^,^30^. Accordingly, assessment of the associations between weather conditions and RTIs on a regional level in developing countries, where the contribution of weather factors to traffic accidents have not been well understood, is essential to the development and implementation of relevant prevention and control strategies, which has been advocated by numerous studies^16^,^21^,^29^,^31^.

The purpose of the present study was to investigate the long-term trends and seasonality of RTIs, and quantify the contributions of meteorological factors to RTI-related cases treated at a tertiary level hospital in Shantou city, China. To our knowledge, this is the first study conducted specifically to explore the association between weather conditions and RTIs in China. Findings from this study will promote a better understanding about the effects of weather factors on RTIs, and have the potential to contribute to improvements in the performance of transportation system, road infrastructure design, education programmes for prevention of RTIs, and weather-responsive traffic management strategies under different weather conditions in the future.

## Results

### Descriptive statistics

Table 1 summarizes the descriptive statistics of monthly RTI cases and monthly mean meteorological variables during the study period. It should be emphasized that there were no missing values in this dataset. In total, 156-month study period and 11263 RTI cases were observed, and the monthly RTIs varied from 17 to 129. The data may be long and large enough to investigate the the contributions of meteorological factors to RTI cases.

### Long-term trends and seasonality

Figure 3 illustrates the time trends and seasonal variations of RTI cases along with changes in meteorological factors. The number of RTI cases has not increased obviously during the study period, and RTIs follow a seasonal pattern with more cases during summer and winter months. The distribution of RTIs generally matched the fluctuations in temperature, sunshine hour and wind speed, presenting similar or converse trends.

### Correlation analysis

Normal distribution Q-Q plots showed that RTIs, relative humidity, wind speed and sunshine hour were general normally distributed, while the others were not (Figure S3). The results of correlation analysis between RTIs and meteorological variables are presented in Table 2, and the cross-correlations of meteorological variables were presented in Table S1. For the RTI cases, there are positive correlations with temperature (r_s_ = 0.21, *P* = 0.008) and sunshine duration (r_p_ = 0.19, *P* = 0.017), while negative association with wind speed (r_p_ = −0.28, *P* < 0.001), but no statistical significance was observed with the remaining variables. Although the correlations between RTIs and the three meteorological variables are relatively weak, the correlation coefficients are statistically significant (*P*＜0.05), implying some dependence of monthly RTIs on meteorological variables.

### Multiple linear regression analysis

Meteorological parameters that were significantly correlated with RTIs were investigated further using step multiple linear regression analysis. Results of the regression analysis showed that monthly average temperature, sunshine hour and wind speed fitted a linear model (Adjusted R^2^ = 0.1993, *P* < 0.001), with the regression coefficients of 0.65 (t = 2.36, *P* = 0.019), 2.23 (t = 2.72, *P* = 0.007) and −27.66 (t = −5.67, *P* < 0.001), respectively (Table 3). The results indicated that about 19.93% of the variation in the number of RTI cases could be accounted for by the combined influence of temperature, sunshine hour and wind speed.

### Regression model diagnosis

Collinearity test found that the VIF values were far less than 10. Skewness/Kurtosis tests for normality of the error terms of the fitted regression model showed that adjusted χ^2^ = 5.19, *P* = 0.0745 (*P* > 0.05), and Breusch-Pagan/Cook-Weisberg test for heteroskedasticity of the error terms with a result that χ^2^ = 2.06, *P* = 0.1513 (*P* > 0.05). For the final regression model, Fig. 4 displayed the results of regression diagnosis and residual analysis. The general well goodness of fit between temperature, sunshine duration, wind speed and RTI cases were observed, the residual plot implied that the error terms with mean zero and constant variance, and compared with the normal curve, kernel density estimate for the error terms presented a general normal distribution.

## Discussion

In the current study, we found that more RTIs from the tertiary level hospital in Shantou city occurred during summer and winter months, and the number of cases each month rose with increasing temperature and sunshine hours, while wind speed was negatively correlated with RTIs. In the regression analysis, temperature, sunshine hour and wind speed were retained in the final model, and accounted for approximately 19.93% of the occurrence of RTI cases.

**Figure 1:**
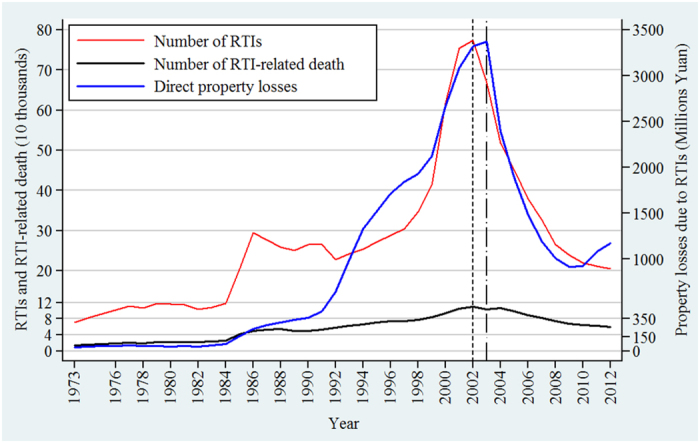
The situation and direct property losses of RTIs in China, 1973–2012.

With rapid economic development the number of motor vehicles in China has increased by 16.9% per year on average, from 42 million in 1997 to 145 million in 2006^18^,^32^, and the kilometres of roads have increased up to around 4.35 million^13^,^33^. Similar trends could also be observed in Shantou city (Figure S1). Between 1985 and 2005, the population-based rate of RTI-related death almost doubled, from 3.9 per 100,000 to 7.6 per 100,000, making RTIs as a “primary public hazard” to human health in China^15^,^34^. There was a slight downward trend since 2004 in RTI-related death (Fig. 1), but taking into account the total lengths of roadway, large number of population and automobiles, and huge direct property losses, China is still facing an arduous challenge in the control of RTIs^13^,^16^,^17^,^32^,^35^.

There are no obvious long-term trends in the number of RTIs treated at the studied hospital (Fig. 3). The reasons may be partly attributable to traffic congestion in urban areas, which has been becoming a common phenomenon in recent years and could lead to lower traffic speed. Moreover, in 2004, China implemented a new road traffic safety law, and since then many preventive measures that had been proved successful in developed countries, such as standardizing warning signal systems, enforcing speed limits and improving conditions of roadway surface, have been introduced to deal with RTIs^18^,^34^.

According to the definition of *China Meteorological Administration*, China has four distinct seasons: spring (Mar–May), summer (June–August), autumn (September–November) and winter (December–February). In the present study, relatively more RTIs were prevalent during summer and winter months (Fig. 3). Summer season was characterized by more extremes of temperature and daily hours of intense sunlight, which could impose heat stress on drivers, cyclists and pedestrians. It is possible that heat stress may affect the wellbeing and safe performance of traffic participants, including get tired easily, cannot concentrate on driving and more fluctuation of emotion, and accordingly increase the risk of injury per kilometer travelled^36^,^37^. In addition, it should be noted that traffic volumes and activity are usually higher in summer months, because more and more people tend to spend their leisure time of summer vacation traveling around in recent years in China (http://www.cnta.gov.cn/)^38^,^39^. Another peak period of RTIs in winter months was also observed in the current study, which is inconsistent with some other studies^31^,^37^,^40^. The possible reason may be due to the Chinese traditional Spring Festival holidays in this season, namely Spring Festival travel season, when the traffic volumes and passenger counts are emerging in large numbers, and bring about extremely high overloading, traffic activity and RTIs^36^,^41^. In winter season, others have noted the incidence of RTIs rises strikingly when temperatures fall below freezing, this may be another reason for the seasonal pattern of RTIs in the current study^42^.

**Figure 2:**
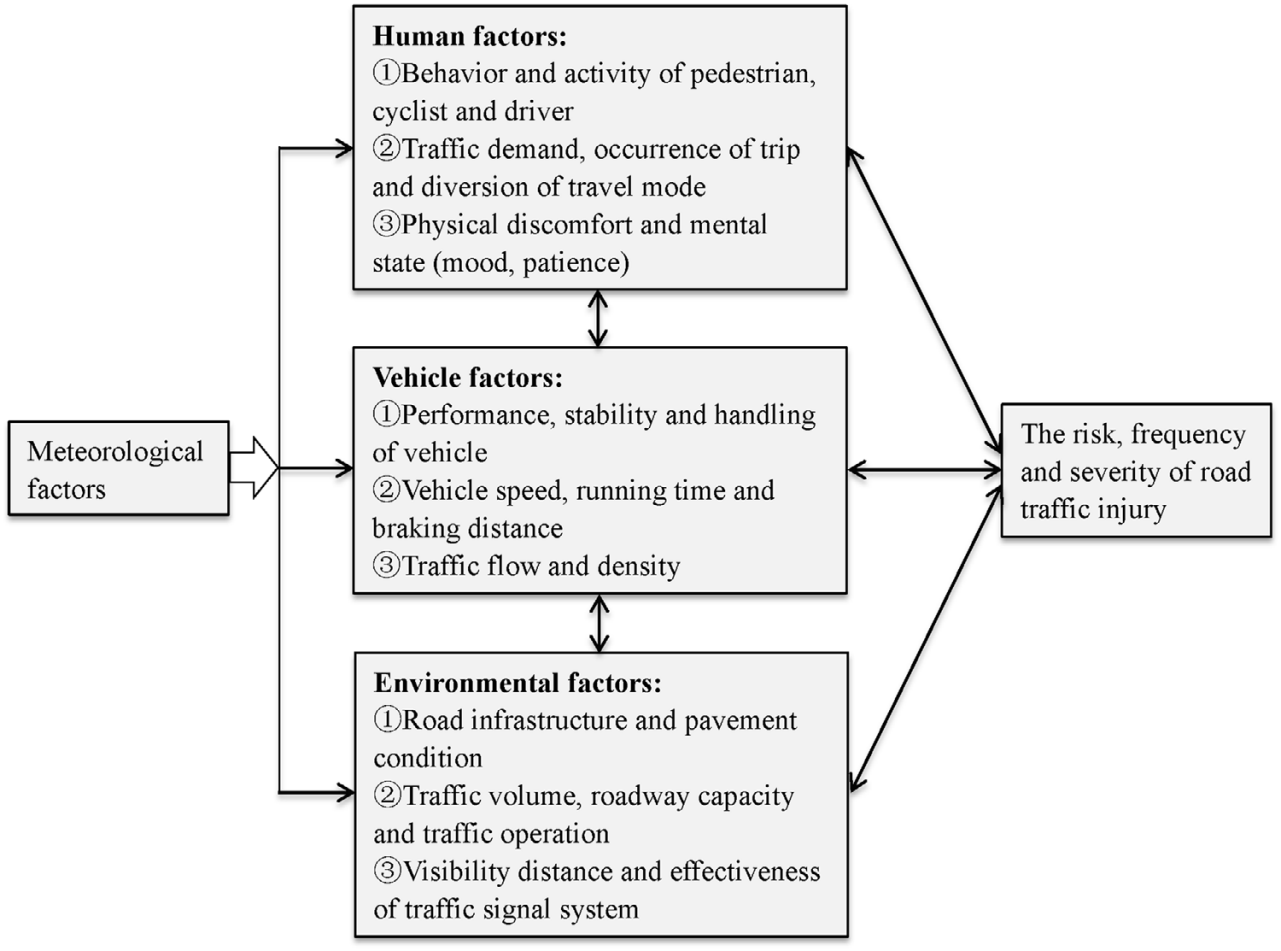
Relationships between weather conditions, road traffic injury and the interactive factors of the famous “Haddon matrix model” (namely human, vehicle and environmental factors).

Meteorological factors can affect RTIs in a variety of ways (Fig. 2). As mentioned above, increasing temperature may imply a perceptible threat to physical comfort and safe performance of drivers, cyclists and pedestrians. It has been argued that the occurrence of RTIs in summer has some correspondence with aspects of the concept of a thermal comfort range, and when temperatures are beyond the upper limit of this range, the participants of road traffic may appear reaction times increase, less good judgement and irritability^37^,^43^. In addition, temperature may result in increasing effects on traffic intensity, activity and volumes, which could escalate the risk of RTIs indirectly^23^,^43^. For example, findings from Bergel-Hayat *et al.* indicate that 1 °C of additional average temperature increases the number of injury accidents in the same month by 1–2%^44^. In accordance with previous studies, the results of correlation analysis and regression model in this study both suggested that temperature was positively associated with RTIs.

Rainfall is a common weather parameter that has been frequently considered in previous studies, and some findings report that rainfall is generally associated with increased number of RTIs^21^. By and large, rainfall influences RTIs mainly through three ways: firstly, the friction of the road surface in contact with the tyres of a vehicle is reduced during wet conditions because of rainfall, leading to the requirement for greater stopping distances; secondly, wet conditions caused by rain may increase the difficulty of vehicle handling, and decrease vehicle performance simultaneously; and thirdly, visibility may be restricted or disturbed during rain^23^,^45^,^46^. However, slippery road surface and poor visibility caused by rainfall can influence RTIs differentially, and some other studies have suggested non-significant or even negative correlations between rainfall and RTIs^44^,^47^,^48^. Similarly, there was no significant association between rainfall and RTIs being observed in our study (r_s_ = −0.023, *P* = 0.77). The discrepancy can be explained by the following possible reasons: first, adverse weather (rainfall) may increase the caution and tendency of drivers to reducing speeds, maintaining safe spacing, and driving more carefully; second, during rainy weather, may be there is a decline in the number of pedestrians and traffic activity, and some participants of road traffic may choose public transport or refrain from going out; lastly, since Shantou city has a regular and obvious rainy time of year, known as plum rain season, which usually occurs around the same months every year, consequently, the adaption and acclimatization of the local population may play a role in determining the effects of rainfall on RTIs^21^,^23^,^46^,^49^.

**Figure 3:**
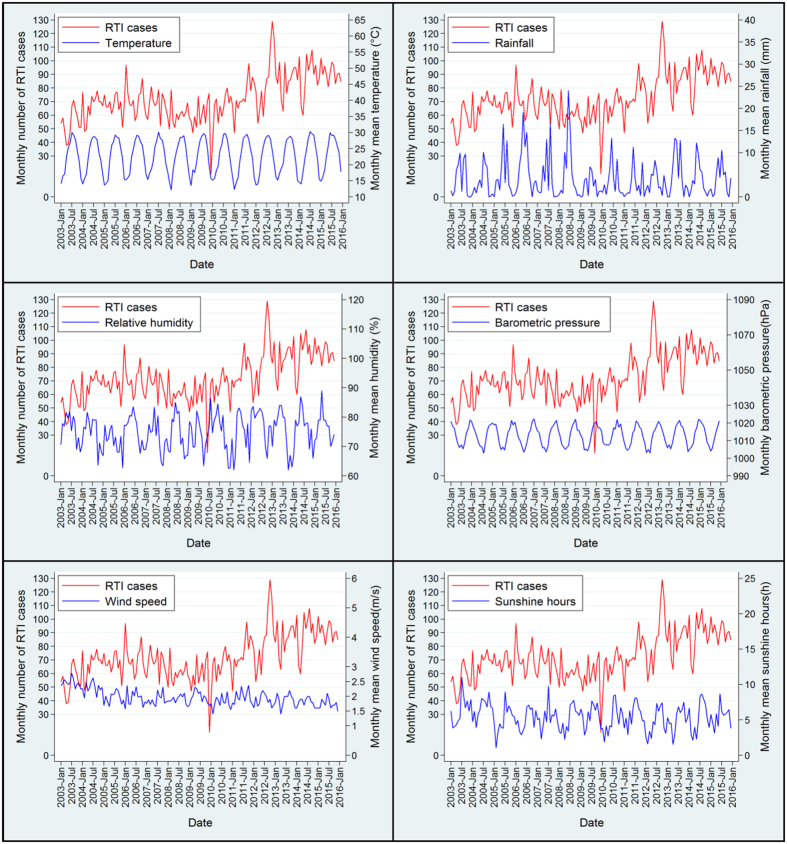
Trends and characteristics of the monthly time series between January, 2003 and December, 2015, for road traffic injury cases (RTIs), temperature, rainfall, relative humidity, barometric pressure, wind speed and sunshine hours.

Some peak periods of RTIs were observed specifically during summer seasons which were characterized by more daily hours of sunlight (Fig. 3). Sunlight can make the driver’s task more difficult due to more dazzle^37^. But a more important factor is likely to be the average increase in traffic activity and intensity associated with longer sunshine hours, which could impose a negative effect on road safety^23^,^27^,^50^. Our findings are consistent with those from previous studies, pointing out that extra sunshine hour increases the number of RTIs^21^,^27^.

The effect of wind speed on RTIs is not extensively explored in literatures, and evidence about the effect is usually inconsistent. A handful of studies propose that wind speed has a positive relationship with RTIs, since high winds can make controlling a vehicle difficult, cause visibility restrictions and lane obstructions by blowing dust, snow or other debris across roadways, and decrease vehicle stability, which accordingly increase the risk of road accidents^22^,^27^,^45^,^51^. However, in the present study, a negative association between wind speed and RTIs was observed in both correlation analysis and the regression model, in contrast to studies stated the opposite, the discrepancy requires some explanation. High winds may not only reduce traffic intensity, activity and roadway capacity, but also diminish the number of pedestrians and cyclists, and hence decrease the number of RTIs^23^,^49^,^51^. Moreover, as a coastal city where high wind is a frequent natural phenomena, local population of Shantou may have adapted to the weather conditions by taking possible compensated actions, such as postponing or canceling discretionary trips, changing travel route and destination, or switching modes of travel, which may be enough to counterbalance the adverse effects of wind on RTIs^23^,^27^,^49^.

Relatively few studies have specifically focused on the effects of barometric pressure and humidity on RTIs^21^,^22^. In the current study, there were not statistically significant associations between barometric pressure, relative humidity and RTI cases.

**Figure 4:**
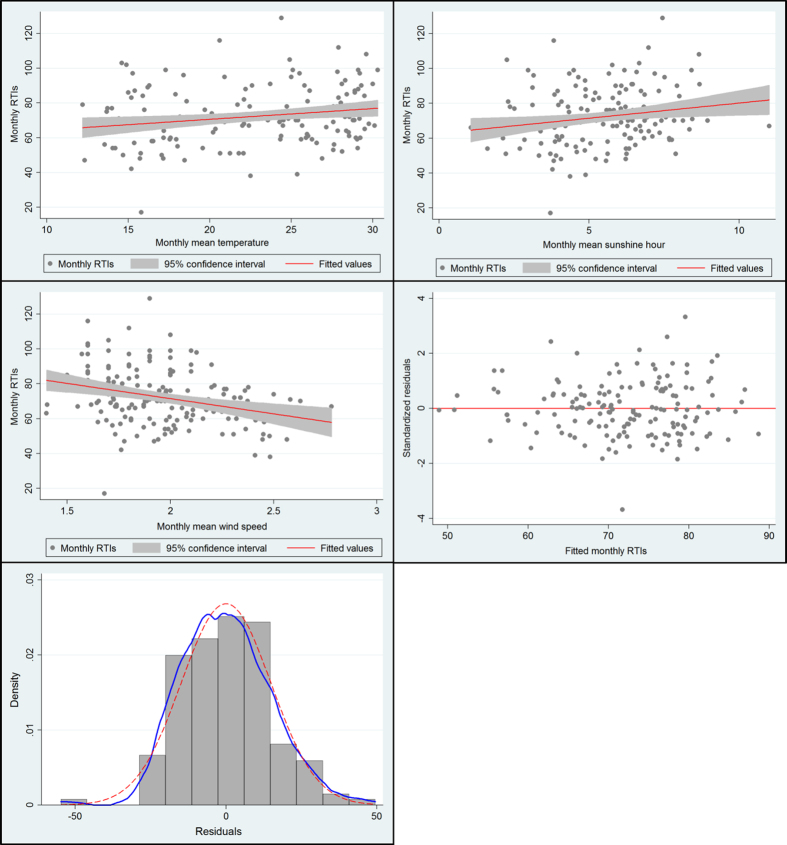
Diagnosis of the regression model (goodness of the fit).

VIFs could measure how much the variances of the estimated regression coefficients are inflated as compared to when the predictor variables are not linearly related^23^. Thus, VIFs are usually employed to evaluate the level of multi-collinearity, and the largest VIF value of all predictor variables is chosen as the indicator for the extent of multi-collinearity^23^,^52^. In this study, all the VIF values were far less than the given criterion 10 (Table 3). In the fitted regression model, heteroskedasticity and normality tests indicated that the error terms was normally distributed with constant variance (homoscedasticity). Regression diagnosis, residual plot and kernel density plot (Fig. 4) also suggested that the assumptions for multiple linear regression are generally satisfied, implying the coefficients of the final regression model are unbiased and have minimum variance among the included estimators.

This study has a number of strengths to support the credibility and prove the value of our findings. Firstly, to the best of our knowledge, this is the first study conducted specifically to investigate the contributions of meteorological factors to the occurrence of RTIs in China. The final regression model found that 19.93% variation in the number of RTIs can be attributable to the combined effects of temperature, sunshine hour and wind speed, which is in accordance with previous studies^26^,^27^,^44^,^49^. Secondly, in this study, all RTI cases were collected from a tertiary level hospital, the first affiliated hospital of Shantou University Medical College, that introduced electronic medical records coded according to ICD-10 in 2002, and since then has applied strict quality management to medical information. Thus, the general problems of misclassification and error-reporting are likely to be minimized. Thirdly, this study was based on 156-month study period and 11263 RTI cases, and all data elements are complete without missing values, therefore, the current study may be enjoy enough statistical power to detect the associations. Finally, the findings of our study may provide evidence support and reasons to the selection of mortality indices when investigate the temperature-mortality relationships. To date, numerous studies choose all-cause mortality or non-accidental death as the mortality indices without explaining the reasons for choosing this over another when they examine the exposure–response association between temperature and mortality^53^,^54^,^55^,^56^. According to the present study, as the leading cause of accidental mortality in China, only 19.93% RTIs from the studied hospital could be accounted for by weather factors, thus, the real temperature-mortality relationships would be underestimated or even misjudged when the accidental deaths (mainly RTI-related) were considered and all-cause death was set as the mortality indicator to investigate the temperature-mortality association^15^,^16^,^57^.

Some limitations of our study should also be acknowledged. Although the first affiliated hospital of Shantou University Medical College is one of the only two tertiary level hospitals in Shantou city, there are still other 70 around hospitals and medical clinics in Shantou region. Some RTI sufferers may seek medical advice in other hospitals, and those who only sustain minor injuries are likely choose to visit a nearby clinic^31^. Therefore, we acknowledge that RTI cases used here may not be representative of all RTIs in Shantou city, the associations with meteorological factors may not apply to the minor injuries, in terms of the influence of weather, and the generalisability of our findings in other regions or studies should be with cautious. Second, other potential risk factors such as the effects of holiday and the day of week were not considered in the regression model, which have been suggested somehow responsible for the occurrence of RTIs in previous research^23^,^37^,^58^. Third, the potential lag effects of some meteorological variables (e.g. rainfall) on RTIs were not taken into account in our analysis, which have been proposed by previous study^21^. Fourth, it is plausible that the contributions of meteorological factors to the occurrence of RTIs could be modified and confounded by other human, vehicle and environment factors, such as behavior and activity of pedestrian, vehicle speed, traffic flow and density, traffic volume, and the number of passenger kilometres. However, in the regression model, we did not adjust for these variables as covariate, because the data were not available in China, especially for the province/city and monthly/daily level. Last, in order to observe the impact of weather conditions on RTIs and to investigate the long-term trends and seasonality of RTIs completely, monthly values were selected as the study variables in our study. Several researchers have warned for biases being introduced by modelling RTIs at high level of aggregation, because they are less suitable for measuring weather influences due to oversimplification, the short term and acute effects of some extreme weather, and cannot pick-up the effects of traffic volume patterns and holidays easily^27^,^58^. However, it is not usually appropriate to perform linear regression analysis using daily counts, since the model requires a large number of daily accident counts, while if not, the errors would not be normally distributed, and may result in misjudgement of the real relationships^44^. Besides, monthly studies could describe the trends and seasonal variation of RTIs more accurately while retaining the advantages of studies on a yearly basis^27^.

## Conclusion

Substantial theoretical and common sense reasons could be offered to explain why meteorological factors can play a role in the occurrence and severity of RTIs. In the present study, it is worthwhile to point out that summer and winter seasons are the relatively peak months of RTIs in Shantou city, and concerning temperature and sunshine hour, the effects are positive on RTIs, while wind speed is negatively correlated with RTIs. According to the regression analysis, temperature, sunshine hour and wind speed were significantly associated with RTIs, and were predictors of up to 19.93% variation in the number of monthly RTI cases from the studied hospital in Shantou city.

As one of the countries with the highest number of traffic accidents, it should be emphasized that systematically analyzing RTIs from different perspectives by applying various methods, to evaluate the situation of RTIs and to reduce the occurrence of RTIs, will still be a arduous challenge for China in the coming years. Findings of this study can help us better understand the relationships between meteorological factors and RTIs, and to contribute to the development and implementation of regional level evidence-based appropriate weather-responsive traffic management strategies under different weather conditions in the future, which have the potential to improve the proactive safety management and traffic service systems on a local basis, and ultimately reduce the health risk and disease burden of RTIs in China.

This exploratory study, may be improved in future by collecting daily RTI data and information (disaggregated according to severity) from more hospitals, traffic surveillance and management systems. Controlling for risk exposures by including information of behavior and activity of pedestrian (e.g. the level of exposure), vehicle speed, traffic density, traffic volume, and the number of vehicle kilometres driven in the models has the potential to improve the interpretability of the regression analysis. However, it is only possible to control for the confounding factors if the datasets of relevant exposure factors were available, thus, recommendations for enriching risk exposure databases and real-time surveillance have been made^21^,^44^. Besides, more sophisticated statistical models to examine the lagging effects of specific meteorological variables on a daily basis while controlling for potential confounding factors simultaneously would also enhance the power of the investigation^42^. There is certainly a need for further research that takes these issues into account to explore the effects of weather conditions on RTIs on both national and regional level in China and worldwide.

## Methods

### Data sources

According to the 10th International Classification of Diseases and Causes of Death (ICD-10), RTIs are classified under the V01–V89 codes. In the present study, daily RTI cases (patients admitted to hospital for diagnosis and treatment because of RTI) coded V01–V89 were provided by a tertiary level hospital (the first affiliated hospital of Shantou University Medical College) in Shantou city of China during January 1st, 2003 to December 31st, 2015. The studied hospital is one of the only two tertiary level hospitals in Shantou region, with nearly 110,000 square meters construction area, 59 clinical departments and 1816 hospital beds. This source is less likely to be affected by under-counting and bias than other RTI data bases. Figure S1 showed that the RTI cases from this hospital has a similar rising trend with the traffic mileages of highway (Km) and possession of civil vehicles in Shantou city. Thus, at least to a certain degree, information from this hospital could give an index to the actual situation of RTIs in Shantou city. Daily meteorological data for the matched time periods were obtained from the *China Meteorological Data Sharing Service System* (http://data.cma.cn/site/index.html), including daily mean temperatures (°C), sunshine hour (h), rainfall (mm), relative humidity (%), barometric pressure (hPa) and wind speed (m/s).

For the daily data, small value like zero accounts for 11.9%, 68.4% and 24.0% of daily RTIs, mean rainfall and sunshine hour counts, respectively. It is not usually appropriate to model linear regression by using these kind disaggregated daily counts, because the errors will not be normally distributed, and the data may be too disaggregate to be of enough help to the regression model (Figure S2)^44^. Besides, at an aggregate level, macro studies have the advantage to investigate general tendency (e.g. seasonal variation) in a large territory of city or country, despite the fact that they need much longer study periods to obtain sufficient observations^27^,^59^. For these reasons, and taking the objectives of this study into account, the daily data were aggregated at monthly level to investigate the contribution of meteorological factors to the occurrence of RTIs.

### Statistical analysis

First, in order to characterize the long-term trends and seasonal fluctuations of RTI cases and meteorological factors, time-series curves were graphed. The time-series diagram has been found to be suitable for assessment of time trends and seasonal variation in past research^44^,^60^. Then, the bivariate Pearson’s correlation analysis or Spearman’s rank correlation test were applied to investigate relationships between weather conditions and RTIs, depending on whether the sampling distributions of relevant variables were normally distributed or not (Figure S3). Last, linear relationships between RTI cases and meteorological factors were tested (Figure S4), and RTI cases generally showed linear relations with weather factors, then the associations of meteorological parameters with RTIs were assessed using stepwise multiple linear regression analysis, which was carried out with RTI cases as dependent variable, and the meteorological factors as independent variables.

In theoretical multiple regression model, several assumptions are made about the explanatory variables and the error term. For the sake of figuring out whether all parameter estimates are reliable and stable or not, the Variance Inflation Factors (VIF) were employed to examine the level and severity of multi-collinearity. A maximum VIF value exceeding 10 indicates that the reliability and stability of the parameter estimates are questionable^52^. In addition, other assumptions for regression model like normality and homoscedasticity of error terms were tested. Regression diagnosis and residual analysis were also used to further test whether the fitted model satisfies the assumptions.

All statistical analyses were performed using Stata 12.1 statistical software (Stata Corporation, TX, USA) and SPSS 19.0 software (SPSS Inc., USA). All statistical tests were two-sided and the level of statistical significance was set at 0.05.

## Additional Information

**How to cite this article**: Gao, J. *et al.* The association between meteorological factors and road traffic injuries: a case analysis from Shantou city, China. *Sci. Rep.* **6**, 37300; doi: 10.1038/srep37300 (2016).

**Publisher’s note**: Springer Nature remains neutral with regard to jurisdictional claims in published maps and institutional affiliations.

## Supplementary Material

Supplementary Information

## Figures and Tables

**Table 1:** Descriptive statistics of monthly road traffic injury cases (RTIs) and monthly mean meteorological factors during the study period. Note. SD, standard deviation.

Variables	Percentile						
	Mean	SD	Min	25th	Median	75th	Max
RTIs	72.00	16.79	17.00	60.00	70.00	83.00	129.00
Temperature (°C)	22.57	5.43	12.20	17.20	23.63	27.89	30.30
Rainfall (mm)	4.13	4.50	0	0.87	2.64	6.07	24.0
Relative humidity (%)	75.03	5.77	62.00	70.11	76.62	79.37	89.00
Barometric pressure (hPa)	1012.98	6.62	1002.90	1007.70	1014.26	1018.02	1022.40
Wind speed (m/s)	1.96	0.27	1.40	1.74	1.92	2.12	2.80
Sunshine hour (h)	5.42	1.85	1.10	4.02	5.57	6.72	11.00

**Table 2:** Correlations between monthly RTI cases and meteorological variables.

Meteorological variables	Road traffic injury cases (RTIs)		
	Coefficient (r_p_ or r_s_)	95% CI	*P*-value
Temperature (°C)	r_s_ = 0.21	(0.052, 0.36)	*P* = 0.008^**^
Rainfall (mm)	r_s_ = −0.023	(−0.17, 0.14)	*P* = 0.77
Relative humidity (%)	r_p_ = −0.047	(−0.19, 0.11)	*P* = 0.56
Barometric pressure (hPa)	r_s_ = −0.079	(−0.24, 0.077)	*P* = 0.33
Wind speed (m/s)	r_p_ = −0.28	(−0.41, −0.14)	*P* < 0.001^**^
Sunshine hour (h)	r_p_ = 0.19	(0.048, 0.32)	*P* = 0.017^*^

Note. r_p_ refers to Pearson’s correlation coefficient, and r_s_ refers to Spearman’s rank correlation coefficient. *P < 0.05, **P < 0.01.

**Table 3:** Multiple linear regression analysis for the associations between monthly road traffic injury cases (RTIs) and meteorological factors.

Model	Standardized Coefficients			95% CI of β	Collinearity Statistics	ANOVA Analysis		Adjusted R Square
	β	t	Sig		VIF	F	Sig	
Constant	99.73	10.90	<0.001	(81.65, 117.81)	–	13.86	<0.001	0.1993
Temperature (°C)	0.65	2.36	0.019	(0.11, 1.19)	1.514			
Wind speed (m/s)	−27.66	−5.67	<0.001	(−37.31, −18.02)	1.203			
Sunshine hour (h)	2.23	2.72	0.007	(0.61, 3.85)	1.583			
