# Supplementary material for: The association between meteorological factors and road traffic injuries: a case analysis from Shantou city, China
Source: Sci Rep. 2016 Nov 17;6:37300. doi: 10.1038/srep37300 (PMC5112526; doi:10.1038/srep37300)
Supplement: Supplementary Information [file srep37300-s1.pdf]

Supplementary Materials

The association between meteorological factors and road traffic injuries: a case analysis from Shantou city, China

Jinghong Gao[1], Xiaojun Chen[2, 3], Alistair Woodward[4], Xiaobo Liu[1], Haixia Wu[1], Yaogui Lu[2], Liping Li[2]*, Qiyong Liu[1]*

[1]State Key Laboratory of Infectious Disease Prevention and Control, Collaborative Innovation Center for Diagnosis and Treatment of Infectious Diseases, National Institute for Communicable Disease Control and Prevention, Chinese Center for Disease Control and Prevention, Beijing, China

[2]Injury Prevention Research Center, Shantou University Medical College, No. 22 Xinling Road, Shantou, Guangdong, 515041, China.

[3]First Affiliated Hospital of Shantou University Medical College, Guangdong, China.

[4]School of Population Health, University of Auckland, Private Bag 92019, Auckland, New Zealand.

* These authors contributed equally to this work as corresponding authors.

Correspondence and requests for materials should be addressed to Q. L. (liuqiyong@icdc.cn)

OUTLINE

Title	Page
**Table S1.** The cross-correlations of meteorological variables (Spearman rank or Pearson correlation coefficients). Note: ** $P < 0.01$.	**3**
**Figure S1.** The possession of civil vehicles and traffic mileages of highway in Shantou city, 1990-2014, and the RTI cases from the studied tertiary level hospital (the first affiliated hospital of Shantou University Medical College), 2003-2015.	**4**
**Figure S2.** Box plots of daily RTI cases, mean wind speed and sunshine hour.	**5**
**Figure S3.** Normal distribution Q-Q plots of analysis variables.	**6**
**Figure S4.** Scatter plots matrix of the analysis variables.	**7**

Variables	Temperature	Rainfall	Relative humidity	Barometric pressure	Wind speed	Sunshine hour
Temperature	1.00	0.52**	0.36**	-0.92**	-0.37**	0.59**
Rainfall		1.00	0.73**	-0.67**	0.13	-0.12
Relative humidity			1.00	-0.55**	0.06	-0.24**
Barometric pressure				1.00	-0.38**	-0.39**
Wind speed					1.00	0.38**

Table S1. The cross-correlations of meteorological variables (Spearman rank or Pearson correlation coefficients). Note: ** $P < 0.01$.

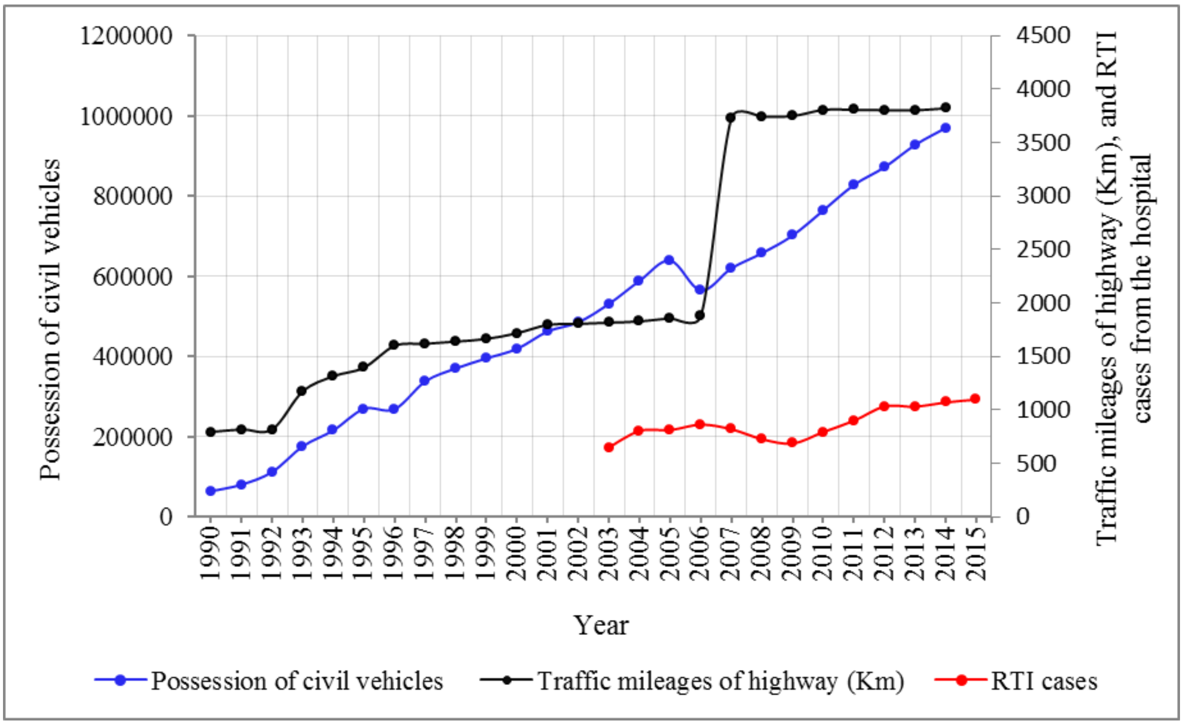

Figure S1. The possession of civil vehicles and traffic mileages of highway in Shantou city, 1990-2014, and the RTI cases from the studied tertiary level hospital (the first affiliated hospital of Shantou University Medical College), 2003-2015.

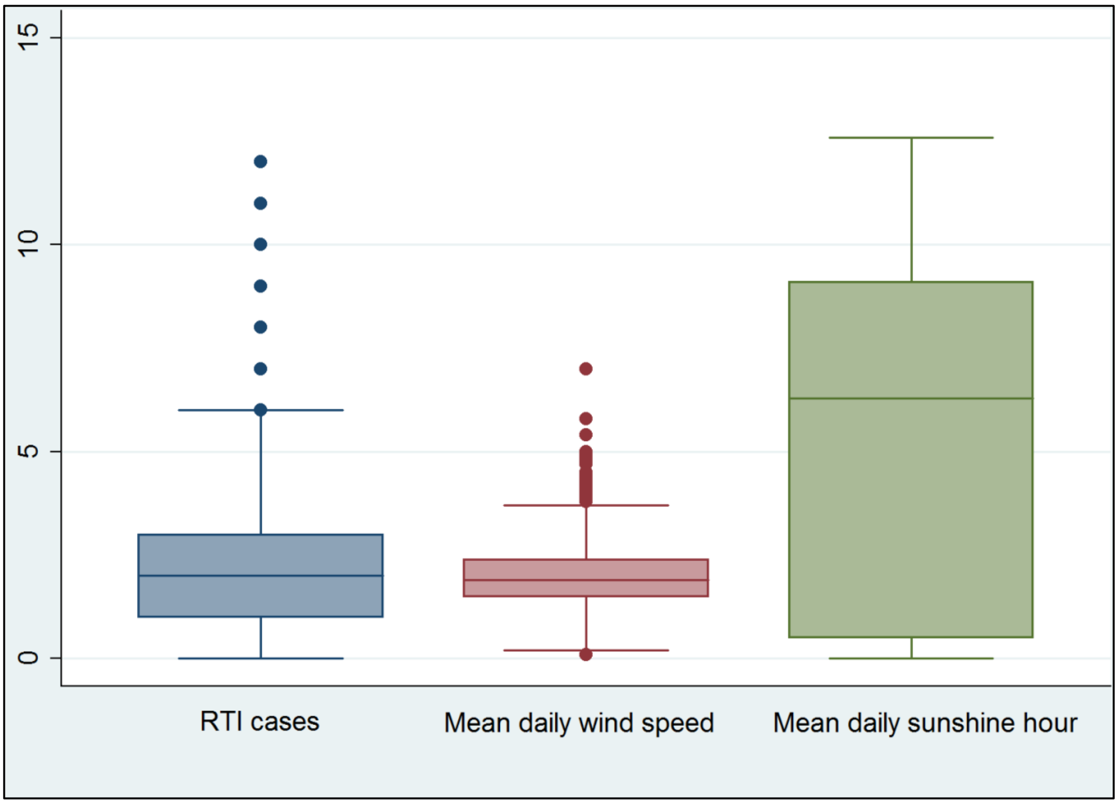

Figure S2. Box plots of daily RTI cases, mean wind speed and mean sunshine hour.

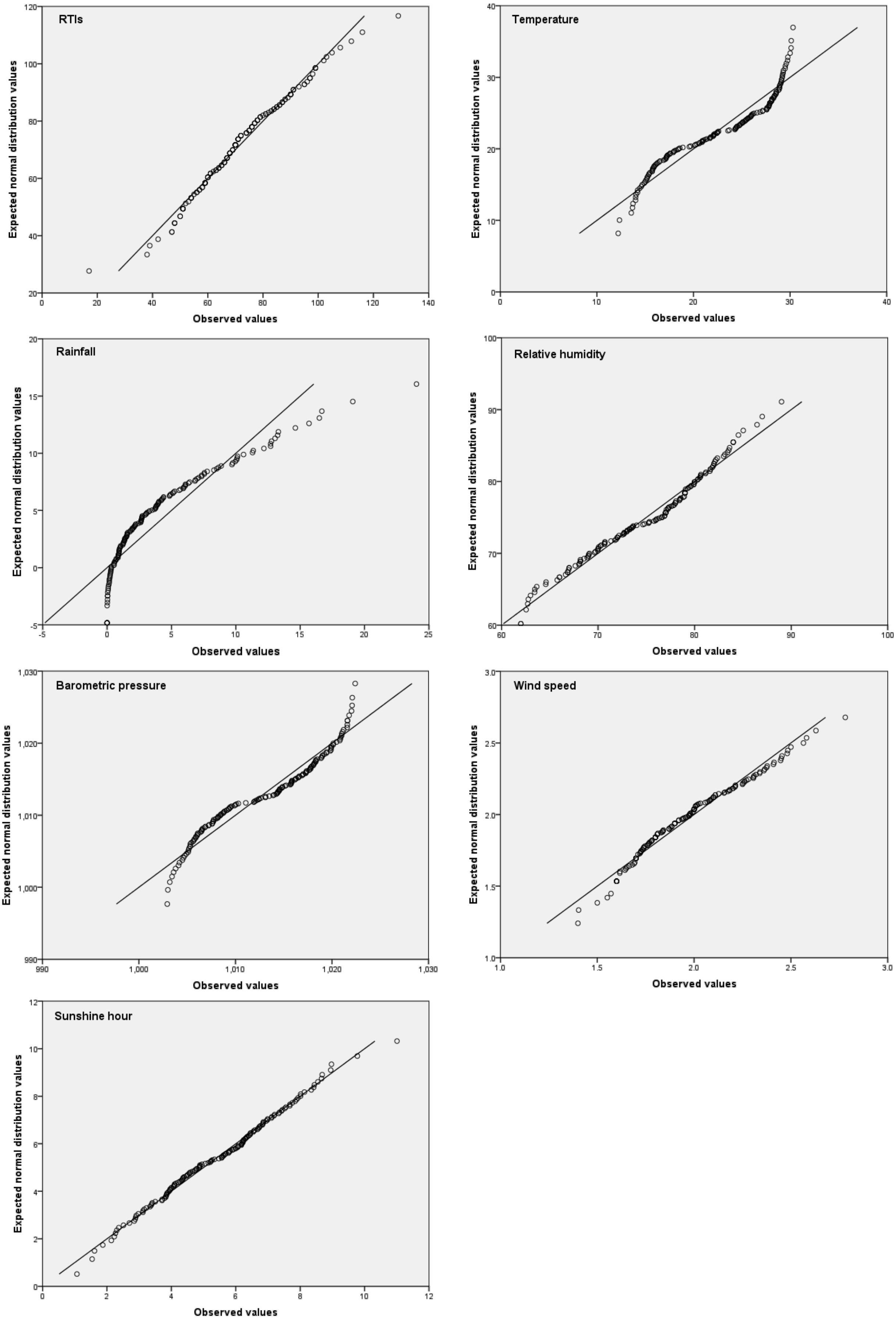

Figure S3. Normal distribution Q-Q plots of analysis variables.

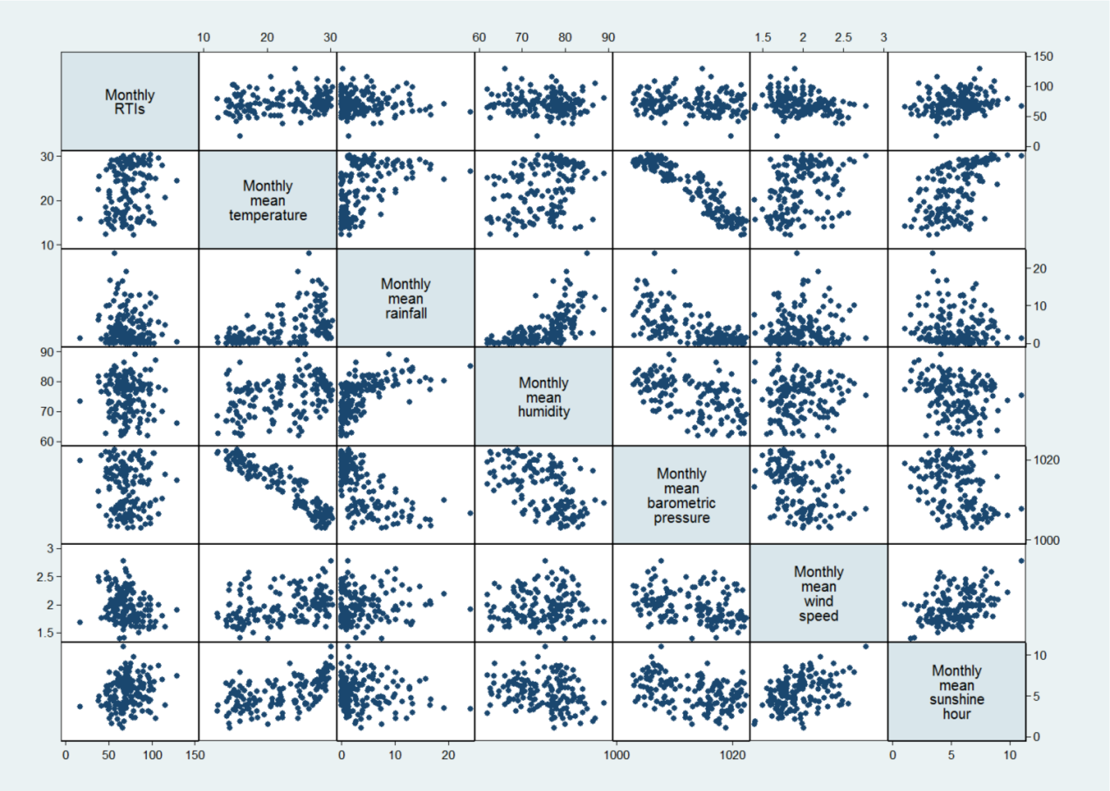

Figure S4. Scatter plots matrix of the analysis variables.
